# Relationship between Basic Neurological Cognition and Social Cognition among Allen Cognitive Disability Levels of Acquired Brain Injury

**DOI:** 10.3390/healthcare8040412

**Published:** 2020-10-20

**Authors:** Myoung-Ok Park, Sang-Heon Lee

**Affiliations:** 1 Division of Health Science, Department of Occupational Therapy, Baekseok University, Cheonan-si 31065, Korea; 2 Department of Occupational Therapy, College of Medical Science, Soonchunhyang University, Asan-si 31538, Korea; sangheon@sch.ac.kr

**Keywords:** social cognition, acquired brain injury, neurological cognition

## Abstract

(1) *Background*: There are various cognitive, perceptual, and social problems associated with acquired brain injury (ABI). The Allen cognitive impairment level indicates the degree of cognitive function required for everyday activities. Until recently, there have been no studies on the relationship between basic neurological cognition and social cognitive function according to the Allen cognitive level (ACL). The aim of this study is to identify the relationship between basic neurological and social cognition among Allen cognitive disability levels of ABI. (2) *Methods*: Thirty-four patients with ABI were identified. Cartoon Intention Inference Task (CIIT), Social Behavior Sequence Task (SBST), Korean version Mimi-Mental Status Examination (K-MMSE), and Lowenstein Occupational Therapy Cognitive Assessment (LOTCA)-tests were administered to examine the differences in neurological and social cognitive functions according to each participant’s Allen Cognitive Level Screening (ACLS). (3) *Results*: There were significant differences between K-MMSE, LOTCA, CIIT and SBST results among Allen cognitive levels (*p* < 0.05). There was a linear correlation between K-MMSE (*r* = 0.778, *p* < 0.01), LOTCA-total score (*r* = 0.627, *p* < 0.01), LOTCA-orientation (*r* = 0.470, *p* = 0.01), LOTCA-thinking operation (*r* = 0.341, *p* < 0.05), CIIT (*r* = 0.817, *p* < 0.05), and SBST (*r* = 0.376, *p* < 0.05) and ACL. Stepwise multivariate regression showed that the subscales affecting the ACLS score were SBST (*β* = 0.239, *p* = 0.000) and K-MMSE (*β* = 0.068, *p* = 0.001). The explanatory power of this regression equation, R^2^, was 0.767. (4) *Conclusions*: A significant difference was found in neurological and social cognitive function according to the ACL level of the ABI patient. In addition, there was a linear correlation between the ACLS scores of the ABI patients and the underlying neurological cognitive function and social cognition. The higher the overall functional cognitive level (i.e., the group with higher ACLS scores), and the lower the degree of help required in daily life, the higher both the neurological cognition level and social cognitive level were determined to be.

## 1. Introduction

Acquired brain injury (ABI) refers to damage to the brain that occurs after birth due to a traumatic or non-traumatic incident. Stroke and traumatic brain injury are the main causes of ABI [1]. ABI patients suffer neurological impairments such as motor deficits and sensory loss as well as various cognitive, perceptual, and social disorders [2]. Cognitive function here refers to the ability to manipulate knowledge and information efficiently, and can be divided into neurological cognitive function and social cognitive function [3].

Neurological cognitive impairment refers to loss of working memory, poor executive function, delayed cognitive processing, and impaired attention, and may be a major factor in reducing self-independence in daily life [4]. For example, patients with neurological cognitive impairment after a stroke or traumatic brain injury have difficulty with accurately identify the time and place in which they are currently performing due to a deterioration of functioning [4]. Further, they experience difficulty with normal, daily activities due to a decrease in attention, concentration, and recognition [4].

Social cognition defines the ability to understand cognitive processes related to social behavior or the process of presenting knowledge about relationships with others [5]. It includes the ability to infer and consider others’ thoughts, to share and empathize, to recognize others’ emotions through facial expressions, to recognize the causes of spoken facial expressions, and to recognize social situations and clues related to them [6,7]. This is the key mental process needed to communicate and interact with society and the basis for understanding the intentions and actions of others and taking social actions according to a situation [8,9].

According to the Occupational Therapy Domain and Process Framework-III, a patient’s evaluated social interaction skill among performance skill is equivalent to the work performance skill observed during social interaction [10]. Testing social skills is therefore critical to a complete evaluation. In ABI patients, socially inappropriate behavior, and egocentricity are common. According to Hoffmann et al., ABI stroke patients reported overall impairment in emotional function including self-awareness, thoughts, and understanding others’ feelings [11]. Another study by Grattan et al. compared the difference in empathetic ability between the cerebral injury group and a control group. Their results showed that all patients with right and left hemispheric lesions experienced decreased empathetic ability [12]. Psychosocial problems such as communication issues, impulsiveness, disinhibition, aggressiveness, and perseveration often explain an ABI patient’s lack of interaction and participation [13]. This difficulty with social contact and initiation effects the patient’s ability to carry out leisure activities, work, and social activities [14].

According to the Allen cognitive disability model, the degree of independence in performing functional activities by cognitive impairment varies [15]. Occupational therapists can assess this degree of difficulty through screening for the Allen cognitive level (ACL), a test made up of scores from level 1 to 6 (Appendix A: Table A1). While there have been studies on the relationship between ACL, neurological level, and activities of daily living [15], there has not been a study to determine the relationship between social cognition and the functional cognitive level of ABI patients. In this study, we aim to clarify this relationship.

## 2. Materials and Methods

### 2.1. Participants

This study is a cross-sectional observation study. The thirty-four ABI patients who were receiving treatment at a rehabilitation hospital in Seoul participated in the study. Subject participated in this study from January to October 2017. General information was obtained through interviews, and social cognitive function and basic neurological cognitive function tests were performed. Prior to the commencement, the study was reviewed and approved by the research ethics committee (No.1040875-201506-SB-026).

The inclusion criteria consisted of inpatients (a) with ABI due to traumatic brain injury or cerebral hemorrhage, (b) who agreed to participate in the study, and (c) who could understand and follow the evaluation instructions. All patients who agreed to participate in the study were scheduled to perform these tests in the occupational and cognitive treatment rooms. During the study design phase, two occupational therapists evaluated the results of the Korean Mini-Mental Status Examination (K-MMSE), Lowenstein Occupational Therapy Cognitive Assessment (LOTCA), Cartoon Intention Inference Tests (CIITs), Social Behavior Sequence Tasks (SBSTs), and Allen Cognitive Level Screening (ACLS) tests. By doing so, errors between examiners were minimized.

### 2.2. Measures

#### 2.2.1. Korean Mini-Mental Status Examination (K-MMSE)

The MMSE is an extensively used diagnostic tool for measuring changes in cognitive function in clinical and research settings, not only for the screening of cognitive dysfunction but also as a tool in epidemiological studies and diagnostic longitudinal studies. The Korean version of the Mini-Mental State Examination (K-MMSE) is a modification of the Mini-Mental State Examination (MMSE) developed by Folstein, USA in 1975. It consists of six items: Orientation, memory registration, recall, attention and calculation, language, and reasoning and judgment. The maximum total score is only 30 points, and the lower the score, the higher the susceptibility to cognitive decline [16].

#### 2.2.2. Lowenstein Occupational Therapy Cognitive Assessment-II (LOTCA-II)

The Lowenstein Occupational Therapy Cognitive Assessment (LOTCA-II) was used to measure the neurological cognitive function of participants. It was developed based on the cognitive model of Luria and Piaget. The sub-items include orientation, visual perception, spatial perception, motor praxis, visuo-motor organization, thinking operation, and attention. The scale of the test for each sub item is worth at least one point from a maximum of four to eight points, and the total test time is about 40 min. From these scores, it is possible to infer problems of cognitive and perceptual information processing ability. In addition, each test session can be divided into two or three sessions to accommodate the patient. (If a test session is divided, this is recorded in the concentration column.) The inter-rater reliability is 0.87–0.97, which makes it a highly reliable tool [17].

#### 2.2.3. Allen Cognitive Level Screening Test (ACLS)

The ACLS, developed by Allen in 1985, was created for the early identification of cognitive function impairment. Each stage is designed to discriminate between levels 3.0 to 5.8. The testing tool consists of a leather plate, two Perma-Lok needles, and two leather straps. The patients are required to complete three stitches using the straps on the plate: running stich, whip stich, and cordovan stitch. The examiner then observes the sewing performance, which can be an indicator of overall cognitive function. An advantage to this method is that the level of functional independence and adaptive ability can be easily discriminated, and there is no limitation due to cultural difference in use [18]. The degree of safety and the level of assistance necessary with daily life can be represented, and an appropriate guide for the caregiver provided (Appendix A: Table A1) [18]. 

#### 2.2.4. Cartoon Intention Inference Test (CIIT)

The Cartoon Intention Inference Test (CIIT) was used to examine the subjects’ social cognitive function. CIIT was mainly developed by Sarfati et al. to evaluate the social cognitive function of schizophrenic patients. The CIIT is composed of six situational story picture cards. Subjects demonstrate an understanding of the situation depicted in the first three comic assignments by selecting the appropriate reaction from among the remaining three response cards. Examples include stories that take place in a public bath, and stories that take place at fishing grounds. Each question is scored out of one point for a maximum score of six points [19].

#### 2.2.5. Social Behavior Sequence Task (SBST)

The second social cognition test used was the Social Behavior Sequence Task (SBST). This test was designed by Kwon et al. mainly to measure the social cognitive ability of schizophrenic patients. Six social situations are presented to suit the circumstances of our environment and culture, and each action consists of nine specific steps. Examples include lunch at a fast-food restaurant, buying shoes at a department store, and requesting remittance from a bank. The test method is to randomly arrange cards in each situation and ask the patient to arrange them in order. If a pair of each story sequence is arranged correctly, it is scored as one point, and when it is arranged incorrectly, it is scored as zero points, for a total possible score of eight points [20].

### 2.3. Data Analysis

The overall score of the subjects’ general characteristics, neurological cognition, social cognition, and ACL was expressed in descriptive statistics of the frequency type. Prior to full-scale data analysis, data normality was confirmed through Kolmogorov–Smirnov and Shapiro–Wilk tests. Sample size was calculated by G-power software version 3.1, with α = 0.05 and β = 0.8. Total sample size was found to be suitable for 34 subjects. 

The correlation between neurological or social cognition and ACL was analyzed through the Pearson’s correlation coefficient. To find the difference between basic neurological cognition and social cognition scores among ACLs, ANOVA was used. Stepwise multivariate regression analysis was conducted to determine the main factors of social cognitive function and neurological cognitive function affecting ACLS. Statistical analysis was produced with SPSS Statistics for Windows, version 20.0 (IBM Corp. in Armonk, NY, USA). The significance of all statistical tests was chosen below the significance level of 5%.

## 3. Results

### 3.1. Demographic Characteristics of Participants

Table 1 shows the general characteristics of all participants. The mean age of the patients was 56.23 ± 1.45 years. In gender, 20 patients (58.8%) were male and 14 patients (41.2%) were female. Mean duration of disease was 3.61 ± 2.25 months. The main cause of ABI was consistent with the most common causes of stroke and traumatic brain injury. Eight patients had suffered infarction (23.5%), eight patients traumatic SDH (subdural hemorrhage, 23.5%), seven patients ICH (intracerebral hemorrhage, 20.6%), six patients SDH (17.6%), and five patients traumatic contusion (14.7%). The mean score of K-MMSE was 21.91 ± 4.47, and the mean score of LOTCA-Π was 77.26 ± 14.82, while the mean ACLS score was a level of 4.28 ± 0.72, the CIIT score was 1.94 ± 1.39, and the SBST score was 4.52 ± 1.63 (Table 1).

### 3.2. Comparsion Basic Congnive Funcion and Social Cognition by ACL

Allen cognitive levels of all subjects were analyzed by categorical data (level 3.03–3.9, level 4.04–4.9, level 5.0–5.8), and neurological cognitive function and social cognitive levels are presented in Table 2. The Allen cognitive level of all participants was 11 (32.4%) at level 3.0–3.9, 15 (44.1%) at level 4.0–4.9, and eight (23.5%) at level 5.0–5.8. The ANOVA analysis showed differences in neurological function and social cognitive function at each of the three ACL levels.

The K-MMSE test showed statistically significant differences between ACL level 3 (3.0–3.9) and 4 (4.0–4.9) and between level 3 and 5 (5.0–5.8), but no statistical significance between level 4 and 5. In the LOTCA test results, there were significant differences among ACL level 3, 4, and 5. In the CIIT test, there were no significant differences between level 3 and 4 or between level 4 and 5, but there was a significant difference between levels 3 and 5. The results of the SBST showed significant differences between ACL level 3, 4, and 5 (Table 2).

### 3.3. Correlations between Basic Neurological Cognition, Social Cognition, and ACLS

Pearson’s correlation analysis was performed to examine the relationship between the ACLS score and both neurological cognitive function and social cognitive function. The linear correlation was strong in K-MMSE (*r* = 0.778, *p* < 0.01), CIIT (*r* = 0.817, *p* < 0.05), and LOTCA-total score (*r* = 0.627, *p* < 0.01). LOTCA-orientation (*r* = 0.470, *p* = 0.01), LOTCA-thinking operation (*r* = 0.341, *p* < 0.05), and SBST (r = 0.376, *p* < 0.05) showed weak linear correlations (Table 3).

### 3.4. Stepwise Multiple Regression between Basic Neurological Cognition and Social Cognition on ACLS

Table 4 shows the results of the stepwise multivariate regression analysis to determine the neurological cognitive function and social cognitive factors affecting the ACLS scores. In order to examine the factors affecting the ACLS score, we treated ACLS as a dependent variable, and performed a stepwise multiple regression with K-MMSE, LOTCA-total score, LOTCA-sub items score, CIIT, and SBST. As a result, the regression equation was ACLS = 1.702 + 0.239 (SBST) + 0.068 (K-MMSE), and the explanatory power demonstrated at 76.7%, which was a reliable level.

## 4. Discussion

We found that the higher the SBST score, the higher the K-MMSE score and the higher the ACLS score of the ABI patients. The SBST can be used to determine if the patient is planning and performing behavioral procedures in a context that requires a variety of social interactions [20]. Kim et al. reported that schizophrenic patients can estimate the problem of social cognition through the SBST test [21]. Based on this previous study, it can be seen that this study showed consistent results. In the correlation analysis, the CIIT score showed strong a correlation, but regression analysis showed that the SBST score change was more influential on the ACLS score. Therefore, it is necessary to judge these two evaluations concurrently when we assess patients’ social cognition.

The average K-MMSE score of 21.91 for the participants indicated a level of mild cognitive impairment [16]. According to their ACLs, all participants were categorized into level 3, 4, and 5 with most found to be among level 4.0–4.9 followed by level 3.0–3.9 and 5.0–5.8, respectively. Basic neurological cognition between these three ACL groups showed a significant difference between K-MMSE- and LOTCA-total scores. As a result of examining these differences between categorized groups by post-hoc test, the K-MMSE total score showed that the ACL 3 group was lower than both levels 4 and 5, suggesting a manual action level (a level of functioning that requires a large amount of assistance in daily life).

In the LOTCA-total score, there was a significant difference between the three groups. The level of the LOTCA-total score gradually increased as the cognitive recovery progressed from level 3 to 5, which is the stage where overall performance and daily independence are secured.

Based on the Allen cognitive disability model, patients at level 3 have an attention span that can be maintained for a maximum of 30 min on activities, and moderate assistance is needed to perform repetitive everyday activities [18]. As the overall cognitive function gradually recovers, level 4 patients perform goal-directed activities and can act with minimal assistance in a structured environment. Level 5 patients experience trial-errors, but new learning becomes possible [18] and the basic cognitive function of the patient is restored. This study corroborated those results. According to Kim et al., the correlation between the MMSE and ACLS scores of patients who had suffered a stroke also showed a positive correlation [22]. As a result of examining differences in social cognition among the ACS group, we found that the difference in ACL was similar to that of neurological cognitive function. In the post-hoc test, the CIIT scores were statistically significant at the level 3 and 5 groups. In addition, the SBST test results showed statistical significance at each level. ACLS and CIIT scores showed a strong linear correlation of 0.8 or more and a linear correlation of more than 0.3 in SBST. This result demonstrates that functional cognitive level and social cognition are related. In addition, it implies that overall cognitive function and ability to perform in daily life are due to the differences in the social cognitive function between ABI patients who need extensive assistance and those who can perform new learning under supervision.

In general, neurological recovery progresses until six months after the onset of ABI. At this time, the patient has greater brain plasticity of nervous system functions that affect cognitive as well as motor function [23,24]. Therefore, intervention at the initial stage is important for successful rehabilitation.

Currently, the cognitive rehabilitation program of patients who have suffered an acute stroke is focused on the recovery of neurological cognitive function [23,25]. True, these factors are the main reason for the improvement of patients’ motor skills and cognitive processing and for promoting recovery [26]. However, according to the results presented in this study, it can be seen that not only neurological cognitive function but also social cognition vary and change according to the stage of functional recovery based on ACL level. Therefore, it is necessary to also provide social cognitive training according to a patient’s functional cognitive level.

Spikman et al. [26] analyzed correlations between social cognition and non-social cognition in patients with traumatic brain injuries. Those results showed that Rey’s auditory verbal learning test was correlated with facial expressions linked to emotion stimuli. Although the measurement tools used in the previous studies are different from the present study, these results are similar.

This study has some limitations. First, the study subjects did not consist only of ABI patients due to pure trauma, but also included stroke patients. However, the characteristics of patients’ social cognition may vary depending on the nature of the disease and the area of brain damage [27,28,29].

Second, the study design was a cross-sectional study. After the initial brain injury, changes with the passage of time were not presented in the stages of functional recovery. Therefore, future studies will need more cohort studies to estimate changes from the beginning of the onset of the disease to the time of recovery. In addition, there is a possibility that the disease prevalence periods of the participants were not same, which had different effects on the results. In a future study, it will be necessary to conduct an experiment with a group of the same prevalence period. Despite these limitations, it is clinically significant that this study also found differences in neurological and social cognitive changes, which reflects the functional recovery of ABI patients. Based on these results, we suggest that various levels of both neurological cognitive and social cognitive rehabilitation training should be considered to assist those recovering from such injuries.

## 5. Conclusions

This study demonstrated that there is a significant difference in neurological and social cognitive function according to the ACL level of ABI patients. In addition, there was a linear correlation between the ACLS score of the ABI patients and the underlying neurological cognitive function and social cognition. The higher the functional cognitive level, and the lower the degree of help required in daily life, the higher the neurological cognition and social cognitive level. Based on these results, it is recommended that a variety of social cognitive rehabilitation programs be developed and applied to ABI patients as well as conventional cognitive rehabilitation training.

## Figures and Tables

**Table 1:** General characteristics of participants (*n* = 34).

Variables	Mean ± SD or *n* (%)	Range
Age (year)	56.23 ± 1.45	13–76
Gender Male Female	20 (58.8) 14 (41.2)	
Prevalence period (month)	3.61 ± 2.25	2.0–15.0
Educational Level Elementary Middle High Graduate	3 (8.8) 6 (17.6) 19 (55.9) 6 (17.6)	
Main Cause of ABI Infarction SDH ICH Traumatic SDH Traumatic contusion	8 (23.5) 6 (17.6) 7 (20.6) 8 (23.5) 5 (14.7)	
K-MMSE	21.91 ± 4.47	12.0–28.0
LOTCA-∏	77.26 ± 14.82	41.0–114.0
ACLS	4.28 ± 0.72	3.20–5.20
CIIT	1.94 ± 1.39	0.0–5.0
SBST	4.52 ± 1.63	2.0–8.0

K-MMSE = Korean version Mini-Mental Status Examination, LOTCA = Lowenstein Occupational Therapy Cognitive Assessment, CIIT = Cartoon Intention Inference Test, SBST = Social Behavior Sequence Task, ACLS = Allen Cognitive Level Screening Test, ABI = acquired brain injury, SDH = subdural hemorrhage, ICH = intracerebral hemorrhage.

**Table 2:** Comparison of basic cognitive function and social cognition among ACLs of participants.

ACLS	(a) Level 3 (3.0–3.9)	(b) Level 4 (4.0–4.9)	(c) Level 5 (5.0–5.8)			
	*N* (%)					
	11 (32.4)	15 (44.1)	8 (23.5)			
	Mean ± SD			F	*p*-value	Post-hoc test **
K-MMSE	17.27 ± 4.36	23.53 ± 2.29	25.25 ± 2.12	19.16	0.000 *	(a) ≠ (b) *, (a) ≠ (c) *, (b) = (c)
LOTCA	64.09 ± 9.74	77.27 ± 7.96	95.37 ± 11.22	25.83	0.000 *	(a) ≠ (b) ≠ (c) *
CIIT	1.27 ± 0.64	1.86 ± 1.24	3.12 ± 1.64	5.59	0.008 *	(a) ≠ (c) *, (a) = (b), (b) = (c)
SBST	2.81 ± 0.60	4.80 ± 1.14	6.37 ± 0.91	33.63	0.000 *	(a) ≠ (b) ≠ (c) *

* *p* < 0.05, ** post-hoc test was performed by Bonferroni method. (a) Level 3.0–3.9; (b) level 4.0–4.9; (c) level 5.0–5.8. K-MMSE = Korean version Mini-Mental Status Examination, CIIT = Cartoon Intention Inference Test, SBST = Social Behavior Sequence Task, ACLS = Allen Cognitive Level Screening Test ACL = Allen cognitive level, LOTCA = Lowenstein Occupational Therapy Cognitive Assessment.

**Table 3:** Correlations among basic neurological cognitive function, social cognition, and Allen cognitive level.

Variables											
	K-MMSE	LOTCA							CIIT	SBST	ACLS
		Total Score	Orientation	Visual-Perception	Spatial-Perception	Motor Praxis	Visual-Motor Organization	Thinking-Operation			
K-MMSE	-										
LOTCA Total score	0.726 **	-									
Orientation	0.645 **	0.467 **	-								
Visual perception	0.255	0.368 *	0.169	-							
Spatial-perception	0.195	0.354 *	0.406 *	0.662 **	-						
Motor praxis	−0.111	0.257	0.374 *	0.558 **	0.741 **	-					
Visual-motor organization	0.206	0.509	0.344 *	0.392 *	0.466 **	0.535 **	-				
Thinking-operation	0.322	0.628 **	0.340 *	0.476 **	0.521 **	0.527 **	0.813 **	-			
CIIT	0.353 *	0.733 **	0.101	0.525 **	0.327	0.311	0.546 **	0.734 **	-		
SBST	0.664 **	0.734 **	0.278	0.023	−0.083	−0.219	0.212	0.247	0.375 *	-	
ACLS	0.778 **	0.627 **	0.470 **	0.074	−0.045	−0.132	0.252	0.341 *	0.817 *	0.376 *	-

* *p* < 0.05, ** *p* < 0.01, K-MMSE = Korean version Mini-Mental Status Examination, CIIT = Cartoon Intention Inference Test, SBST = Social Behavior Sequence Task, ACLS = Allen Cognitive Level Screening Test.

**Table 4:** Stepwise multiple regression basic neurologic cognition and social cognition on ACLS.

Variable	B	SE	β	T	*p*
Constant	1.702	0.318		5.350	0.000 *
SBST	0.239	0.052	0.538	4.639	0.000 *
K-MMSE	0.068	0.019	0.421	3.626	0.001 *
R^2^ = 0.767; Adju-R^2^ = 0.752; F = 13.145; *p* = 0.001					

* *p* < 0.01, K-MMSE = Korean version Mini-Mental Status Examination, SBST = Social Behavior Sequence Task, ACLS = Allen Cognitive Level Screening Test.

## Appendix A

**Table A1:** References on ACL by Allen cognitive disability model.

	Means & Assistance in ADL
Level 1	-Awareness. -Person responds to internal cues only. -A change in level of arousal is a specific response to an external stimulus. -Total assistance is needed.
Level 2	-Postural action. -Person’s awareness is limited to own postural actions (proprioceptive cues). -Lack of awareness of the effects that actions have on objects. -Maximum assistance is needed.
Level 3	-Manual actions in response to tactile cues. -Repetitive actions demonstrate an awareness of material objects. -Lack of awareness of cause and effect, end product, or goal. -Attention span is maximum 30 min. -Moderate assistance is needed for repetitive actions safely.
Level 4	-Goal-directed actions demonstrate an awareness of a familiar end-product but fail to solve new problems or anticipate or correct mistakes. -There is no independent new learning and individual cannot invent new motor actions. -Attention span is usually good for up to one hour. -Minimum Assistance is needed when therapists set up goal-directed activities with tangible results.
Level 5	-Person learns new activities -Person explores the impact of activities on the physical elements of the environment. -Through trial and error, person can learn that activities are transferred to other environments.
Level 6	-No global cognitive impairment. -Person anticipates errors and plans actions to prevent errors. -Trial-and-error problem-solving may be covert, and “good judgment” is demonstrated. -No supervision required.
